# A retrospective review of 10-year trends in general anesthesia for cesarean delivery at a university hospital: the impact of a newly launched team on obstetric anesthesia practice

**DOI:** 10.1186/s12913-020-05314-2

**Published:** 2020-05-13

**Authors:** Takamitsu Ikeda, Atsuko Kato, Masahiko Bougaki, Yuko Araki, Takuya Ohata, Seiichiro Kawashima, Yousuke Imai, Jun Ninagawa, Koji Oba, Kyungho Chang, Kanji Uchida, Yoshitsugu Yamada

**Affiliations:** 1 Department of Anesthesiology and Pain Relief Center, The University of Tokyo Hospital, Tokyo, Japan; 2 Department of Anesthesiology, Sanraku Hospital, Tokyo, Japan; 3 Department of Critical Care and Anesthesia, National Center for Child Health and Development, Tokyo, Japan; 4 Department of Biostatistics, School of Public Health, Graduate School of Medicine, The University of Tokyo, Tokyo, Japan; 5 Department of Anesthesiology, Teikyo University School of Medicine, Tokyo, Japan; 6 Department of Anesthesiology, International University of Health and Welfare, Mita Hospital, Tokyo, Japan

**Keywords:** Obstetric anesthesia, Cesarean delivery, Placenta previa, General anesthesia, Multi-disciplinary collaboration

## Abstract

**Background:**

The indications for general anesthesia (GA) in obstetric settings, which are determined in consideration of maternal and fetal outcome, could be affected by local patterns of clinical practice grounded in unique situations and circumstances that vary among medical institutions. Although the use of GA for cesarean delivery has become less common with more frequent adoption of neuraxial anesthesia, GA was previously chosen for pregnancy with placenta previa at our institution in case of unexpected massive hemorrhage. However, the situation has been gradually changing since formation of a team dedicated to obstetric anesthesia practice. Here, we report the results of a review of all cesarean deliveries performed under GA, and assess the impact of our newly launched team on trends in clinical obstetric anesthesia practice at our institution.

**Methods:**

Our original database for obstetric GA during the period of 2010 to 2019 was analyzed. The medical records of all parturients who received GA for cesarean delivery were reviewed to collect detailed information. Interrupted time series analysis was used to evaluate the impact of the launch of our obstetric anesthesia team.

**Results:**

As recently as 2014, more than 10% of cesarean deliveries were performed under GA, with placenta previa accounting for the main indication in elective and emergent cases. Our obstetric anesthesia team was formed in 2015 to serve as a communication bridge between the department of anesthesiology and the department of obstetrics. Since then, there has been a steady decline in the percentage of cesarean deliveries performed under GA, decreasing to a low of less than 5% in the latest 2 years. Interrupted time series analysis revealed a significant reduction in obstetric GA after 2015 (*P* = 0.04), which was associated with decreased use of GA for pregnancy with placenta previa. On the other hand, every year has seen a number of urgent cesarean deliveries requiring GA.

**Conclusions:**

There has been a trend towards fewer obstetric GA since 2015. The optimized use of GA for cesarean delivery was made possible mainly through strengthened partnerships between anesthesiologists and obstetricians with the support of our obstetric anesthesia team.

## Background

Ensuring safety for women receiving cesarean delivery remains a continuing challenge for anesthesiologists in general hospitals with an obstetric service. With sustained commitment to the mission of dealing with a variety of surgical procedures, the department of anesthesiology also plays a role in providing the highest standard of care during the labor and delivery process, which can be accompanied by potential risks for both the pregnant woman and her baby.

The choice of anesthetic technique for cesarean delivery should be determined with consideration of the degree of emergency in relation to maternal and fetal status and comorbidities, as well as of the difficulty or expected duration of procedures [1, 2]. It is generally accepted that neuraxial anesthesia is the preferred choice over general anesthesia (GA), with advantages including avoidance of the potential complications with the maternal airway and neonatal exposure to anesthetic drugs used during induction and maintenance of GA [3, 4]. Neuraxial anesthesia has the additional merit of allowing a mother to see her baby in the moments after birth, and reducing the need for neonatal respiratory support. On the other hand, however, administration of GA remains the more appropriate option under certain circumstances, especially when there is a perceived lack of time to apply neuraxial techniques [1, 5]. In addition to emergent situations, GA may be adopted in women with a potentially life-threatening pregnancy-related condition such as placenta previa [2], but the indications for GA are complicated by the difficulty of identifying parturients at high risk for obstetric hemorrhage prior to cesarean delivery. Concerns with respect to encountering unexpected difficult maternal airway have been a deterrent to the frequent use of GA in obstetric settings [6].

As one of the largest and most comprehensive university hospitals in Japan, the University of Tokyo Hospital aims to offer a comprehensive obstetrics program for women in pregnancy with approximately 1000 deliveries per year. The growing need for perinatal care services for high-risk pregnancies, along with rising recognition of the anesthesiology subspecialty of obstetric anesthesia, prompted us, in 2015, to assemble an advanced team dedicated to clinical obstetric anesthesia practice. Although the team consists of staff anesthesiologists engaged in both obstetric anesthesia and a wide range of perioperative care, they have been in close partnership with multi-disciplinary professionals to contribute to better management for women before, during, and after childbirth.

Previously, we experienced a case of placenta accreta with intraoperative massive hemorrhage with a blood loss of almost 20,000 mL, which consequently led to the greater adoption of GA for women with placenta previa undergoing cesarean delivery at our institution. However, recent years have witnessed a progressive change in our obstetric anesthesia practice, especially since our obstetric anesthesia team was launched in 2015. The purpose of this study was to investigate the trends in cesarean deliveries carried out under GA, focusing on practices instituted by our obstetric anesthesia team.

## Methods

With the approval of our Institutional Review Board (#2203-(6)), we retrospectively analyzed our clinical database regarding the obstetric anesthetic care and management during 10 calendar years from January 1, 2010 through December 31, 2019. We reviewed the medical records of all parturients who received GA to determine the indications for cesarean delivery that required GA, whether elective, emergent, or urgent. The urgent cases in the current study were defined as corresponding to category 1 cesarean section described in the National Institute for Health and Clinical Excellence guidelines [7].

Interrupted time series analysis was used to evaluate how the launch of our obstetric anesthesia team impacted on our practice after 2015. Quarterly trends in the proportion of GA cases were analyzed to ensure an adequate number of time points both before and after the intervention. Residual autocorrelation was tested using the Durbin–Watson test. A *P* value < 0.05 was considered statistically significant. All statistical analyses were performed using SAS ver. 9.4 (SAS Institute, Cary, NC).

## Results

### Trends overview

**Fig. 1:**
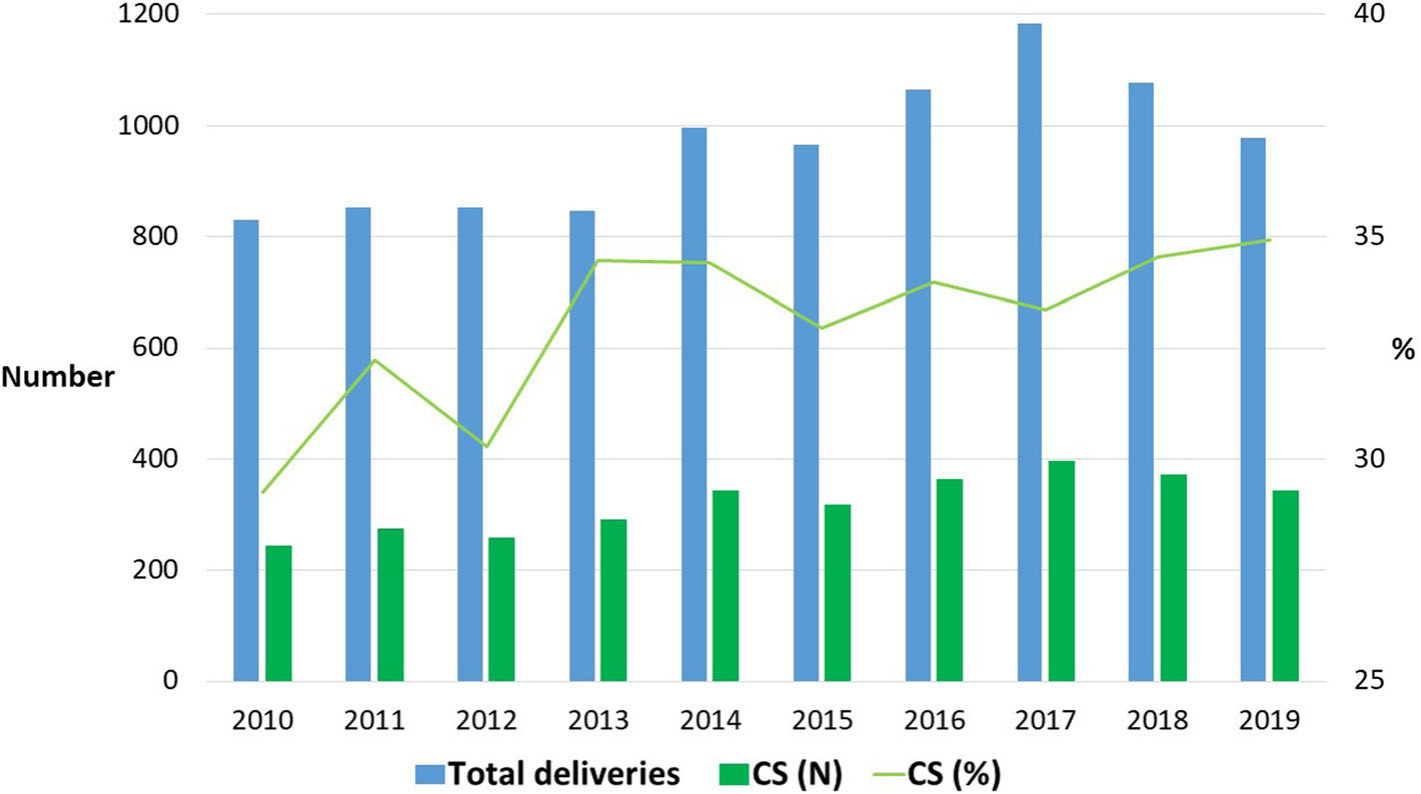
Yearly change in the total number of all deliveries and cesarean deliveries (%) in the years from 2010 to 2019. The annual number of cesarean deliveries has been increasing in concert with an increasing number of total deliveries. In the latest 7 years, approximately one-third of parturients underwent cesarean delivery at our institution

Overall, the annual number of cesarean deliveries was on the rise during the period 2010–2019 at our institution, in tandem with the increasing number of total deliveries (Fig. 1). The proportion of cesarean deliveries remained relatively high throughout the years covered by the study, ranging from 29.2 to 34.9% with a gradual increase over time (Table 1).

**Table 1:** Total number of all deliveries, cesarean deliveries (%), and the number and percentage of cesarean deliveries performed under general anesthesia with classification depending on the degree of emergency

Year	Total deliveries	Cesarean deliveries	GA cases (number / percentage)		
			Elective	Emergent	Urgent
2019	979	342 (34.9%)	15 / 4.4%		
			1 (6.7%)	6 (40.0%)	8 (53.3%)
2018	1077	372 (34.5%)	16 / 4.3%		
			3 (18.8%)	9 (56.2%)	4 (25.0%)
2017	1184	397 (33.5%)	21 / 5.3%		
			4 (19.0%)	8 (38.1%)	9 (42.9%)
2016	1065	361 (33.9%)	21 / 5.8%		
			9 (42.9%)	4 (19.0%)	8 (38.1%)
2015	965	318 (33.0%)	22 / 6.9%		
			6 (27.3%)	12 (54.5%)	4 (18.2%)
2014	997	343 (34.4%)	33 / 9.6%		
			12 (36.4%)	11 (33.3%)	10 (30.3%)
2013	847	291 (34.4%)	36 / 12.3%		
			12 (33.3%)	17 (47.2%)	7 (19.4%)
2012	852	256 (30.0%)	37 / 14.5%		
			16 (43.2%)	18 (48.6%)	3 (8.1%)
2011	853	274 (32.1%)	39 / 14.2%		
			14 (35.9%)	22 (56.4%)	3 (7.7%)
2010	831	243 (29.2%)	27 / 11.1%		
			11 (40.7%)	13 (48.1%)	3 (11.1%)

### General anesthesia for cesarean delivery

**Fig. 2:**
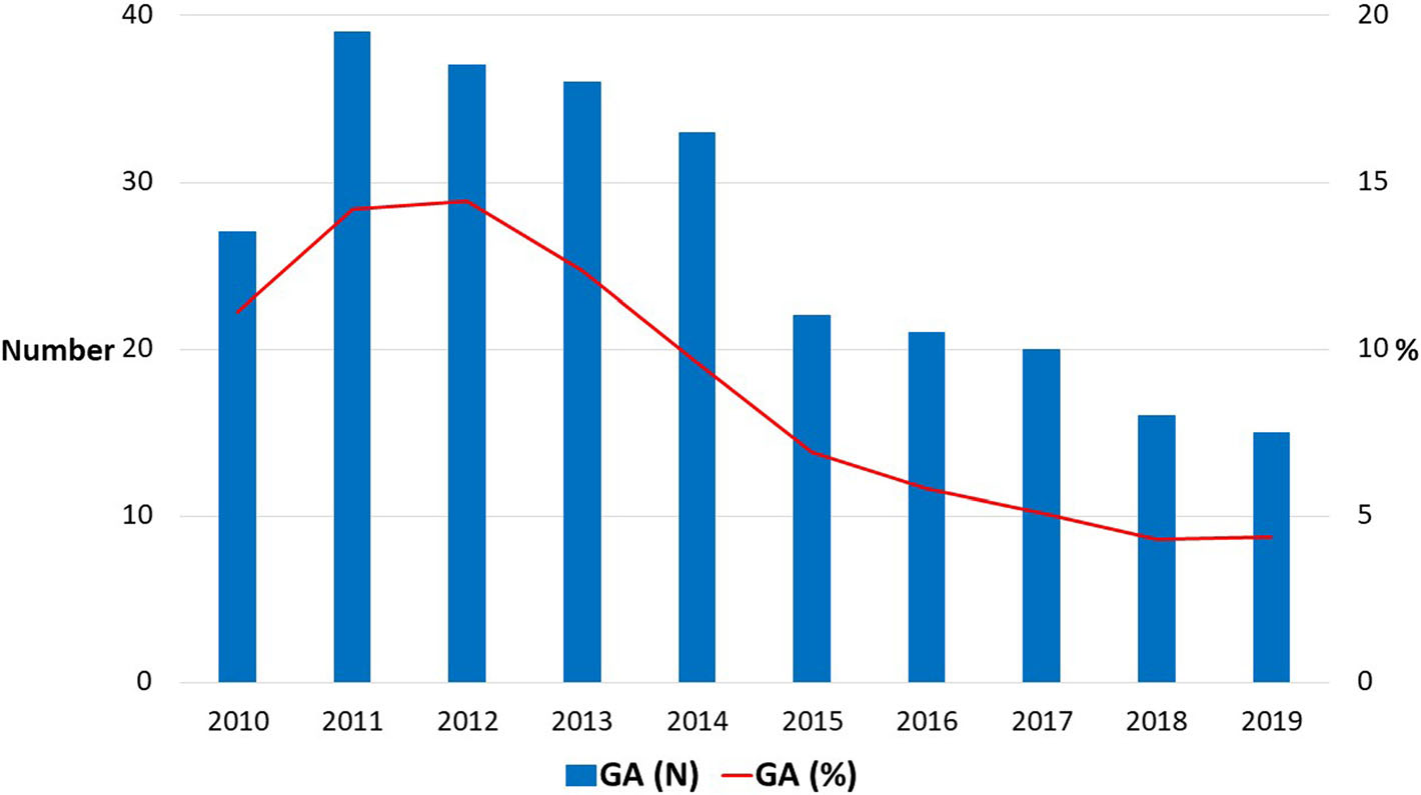
Yearly change in the number and percentage of cesarean deliveries that required general anesthesia. Previously, cases requiring general anesthesia (GA) accounted for more than 10% of the annual number of cesarean deliveries, peaking at 14.5% in 2012. Since then, we have seen a declining trend in the percentage of cesarean deliveries requiring GA, decreasing to a low of less than 5% in 2018 and 2019

As recently as 2014, more than approximately 10% of cesarean deliveries were conducted under GA, with an annual number of 27–39 cases, which peaked in 2011 at 39 cases (Table 1). Since 2015, however, there has been a steady decline in the number of cesarean deliveries requiring GA to 15 in 2019. As a percentage, administration of GA peaked at 14.5% in 2012, but has trended downward, deceasing to less than 5% in 2018 and 2019 (Fig. 2). Elective and emergent cesarean deliveries comprised the majority of all GA cases until recently (Table 1).

**Fig. 3:**
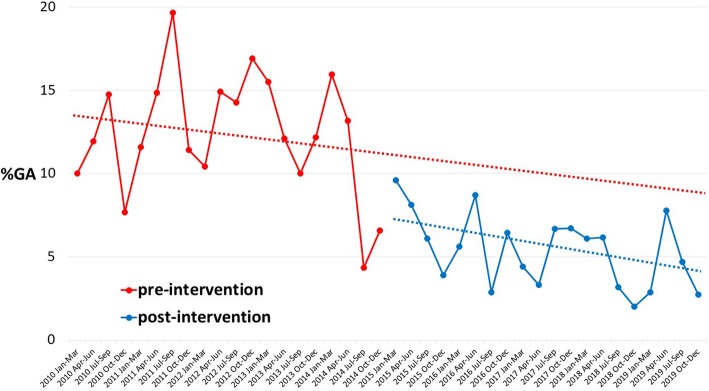
Interrupted time series with comparison between pre- and post-intervention period. The time series of quarterly changes in the percent of cases using general anesthesia (GA) from January 2010 to December 2019 were generated to provide a compassion before and after the intervention. The data show a level change in the proportion of GA cases following the launch of our obstetric anesthesia team in 2015. The blue dots in the post-intervention period 2015–2019 are all positioned below the line (red dashed line) representing the pre-existing trend seen during the pre-intervention period 2010–2014. Interrupted time series analysis revealed that the percent of GA cases was significantly decreased in the post-intervention period (*P* = 0.04). No significant change in slope was found between pre- and post-intervention (*P* = 0.74)

Interrupted time series analysis revealed that there was a significant decrease in the proportion of obstetric GA after the launch of our obstetric anesthesia team in 2015 (*P* = 0.04) (Fig. 3). The graphic provides a visual presentation of decreased use of obstetric GA in the post-intervention period 2015–2019 (blue). All post-intervention data points lie below the trend line extrapolated from the pre-intervention, 2010–2014, data (red). There was no significant change in slope from pre- to post-intervention (*P* = 0.74). The Durbin–Watson statistic showed that there was no evidence of autocorrelation (DW 1.64, *P* = 0.95).

### General anesthesia for pregnancy with placenta previa

**Fig. 4:**
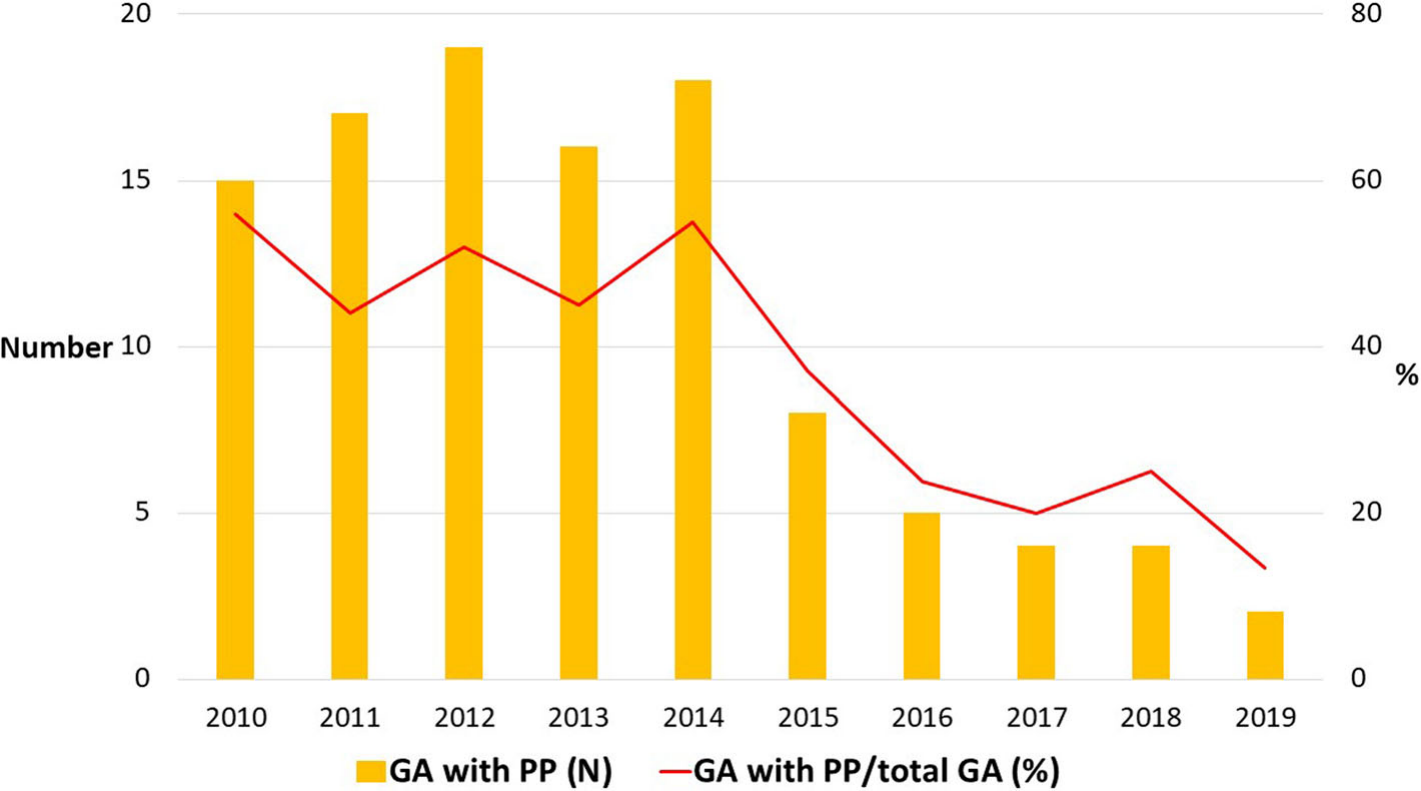
The number and percentage of parturients with placenta previa who received general anesthesia for elective or emergent cesarean delivery in 2010–2019. Before 2015, the annual number of parturients with placenta previa (PP) who underwent elective or emergent cesarean delivery under general anesthesia (GA) exceeded 15, accounting for more than 40% of total GA cases. However, there has been a marked decline since 2015 both in the number and proportion of cesarean deliveries for parturients with placenta previa that were conducted under GA

**Fig. 5:**
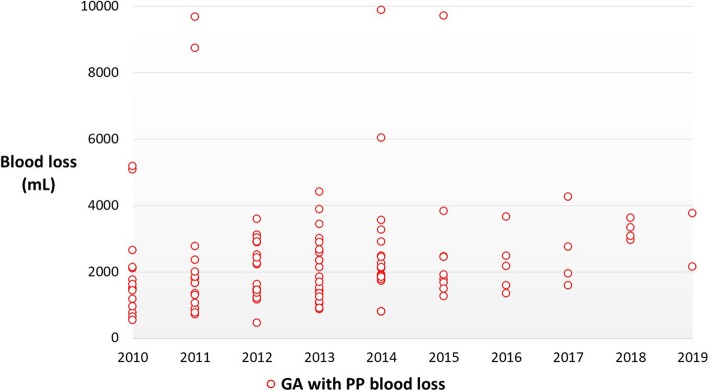
Blood loss during cesarean delivery under general anesthesia in parturients with placenta previa. Each red circle represents a case of placenta previa (PP) conducted under general anesthesia (GA). During the study period, 9 cases with an intraoperative blood loss of more than 4000 mL were recorded

There has been an upward trend in the number of women with placental abnormalities at our institution, exceeding 20 per year (Supplementary Table 1). GA was chosen for pregnancy with placenta previa in the years 2010–2014, a time when placenta previa was the main indication for elective and emergent cesarean deliveries, with proportions ranging from 47.2 to 78.3% (Table 2). However, the annual number of parturients with placenta previa who received GA for elective or emergent cesarean delivery was on the decline (Fig. 4), accounting for 28.6% of the elective and emergent cases in 2019 (Table 2). During the study period, the median blood loss in cesarean deliveries for placenta previa was 1920 mL with an interquartile range (IQR) of 1423 to 2896 mL, although there were 9 sporadic cases with a blood loss exceeding 4000 mL (Fig. 5). Despite these cases of massive hemorrhage, the use of obstetric GA has been on a downward trend in recent years.

**Table 2:** Indications for elective and emergent cesarean deliveries performed under general anesthesia

	2010	2011	2012	2013	2014	2015	2016	2017	2018	2019
Total cases	24	36	34	29	23	18	13	12	12	7
Abnormal placentation	15 (62.5%)	17 (47.2%)	19 (55.9%)	16 (55.2%)	18 (78.3%)	8 (44.4%)	5 (38.5%)	4 (33.3%)	4 (33.3%)	2 (28.6%)
Non-reassuring fetal status	2 (8.3%)	1 (2.8%)	1 (2.9%)	3 (10.3%)	2 (8.7%)	0 (0.0%)	0 (0.0%)	0 (0.0%)	0 (0.0%)	0 (0.0%)
Placental abruption	2 (8.3%)	4 (11.1%)	2 (5.9%)	5 (17.2%)	0 (0.0%)	3 (16.7%)	1 (7.7%)	1 (8.3%)	0 (0.0%)	0 (0.0%)
Threatening uterine rupture	0 (0.0%)	0 (0.0%)	0 (0.0%)	0 (0.0%)	0 (0.0%)	1 (5.6%)	0 (0.0%)	1 (8.3%)	1 (8.3%)	0 (0.0%)
Maternal factors	4 (16.7%)	10 (27.8%)	11 (32.4%)	3 (10.3%)	2 (8.7%)	2 (11.1%)	7 (53.8%)	5 (41.7%)	4 (33.3%)	3 (42.9%)
Fetal factors	1 (4.2%)	3 (8.3%)	1 (2.9%)	2 (7.0%)	1 (4.3%)	1 (5.6%)	0 (0.0%)	0 (0.0%)	0 (0.0%)	1 (14.3%)
Failed spinal	0 (0.0%)	1 (2.8%)	0 (0.0%)	0 (0.0%)	0 (0.0%)	2 (11.1%)	0 (0.0%)	1 (8.3%)	3 (25.0%)	1 (14.3%)
Patient’s request	0 (0.0%)	0 (0.0%)	0 (0.0%)	0 (0.0%)	0 (0.0%)	1 (5.6%)	0 (0.0%)	0 (0.0%)	0 (0.0%)	0 (0.0%)

Maternal factors included threatened premature labor, failure of labor process, hypertensive disorders of pregnancy (HDP), syndrome of hemolysis, elevated liver enzymes, and low platelets (HELLP), impaired placental function, intrauterine infection, and other maternal comorbidities

Fetal factors included diaphragmatic hernia, umbilical cord problems, prolapse of fetal extremity, and other fetal comorbidities

### Urgent cesarean delivery

**Fig. 6:**
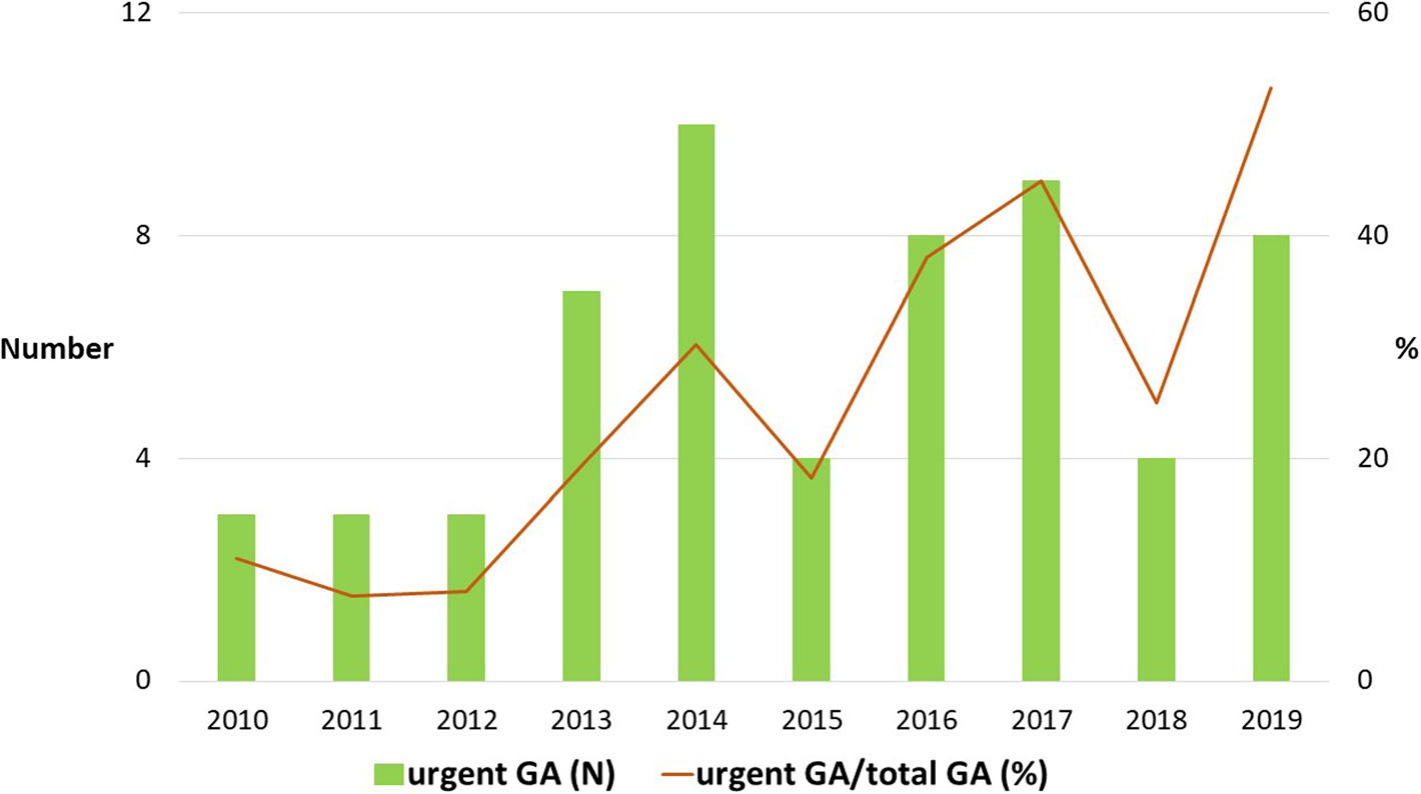
The number and percentage of urgent cesarean deliveries that required general anesthesia in 2010–2019. The annual number of cesarean deliveries that required urgent administration of general anesthesia (GA) remained rather small before 2013. Although varying from year to year, there were annually about 7 urgent cases in the past 7 years

**Fig. 7:**
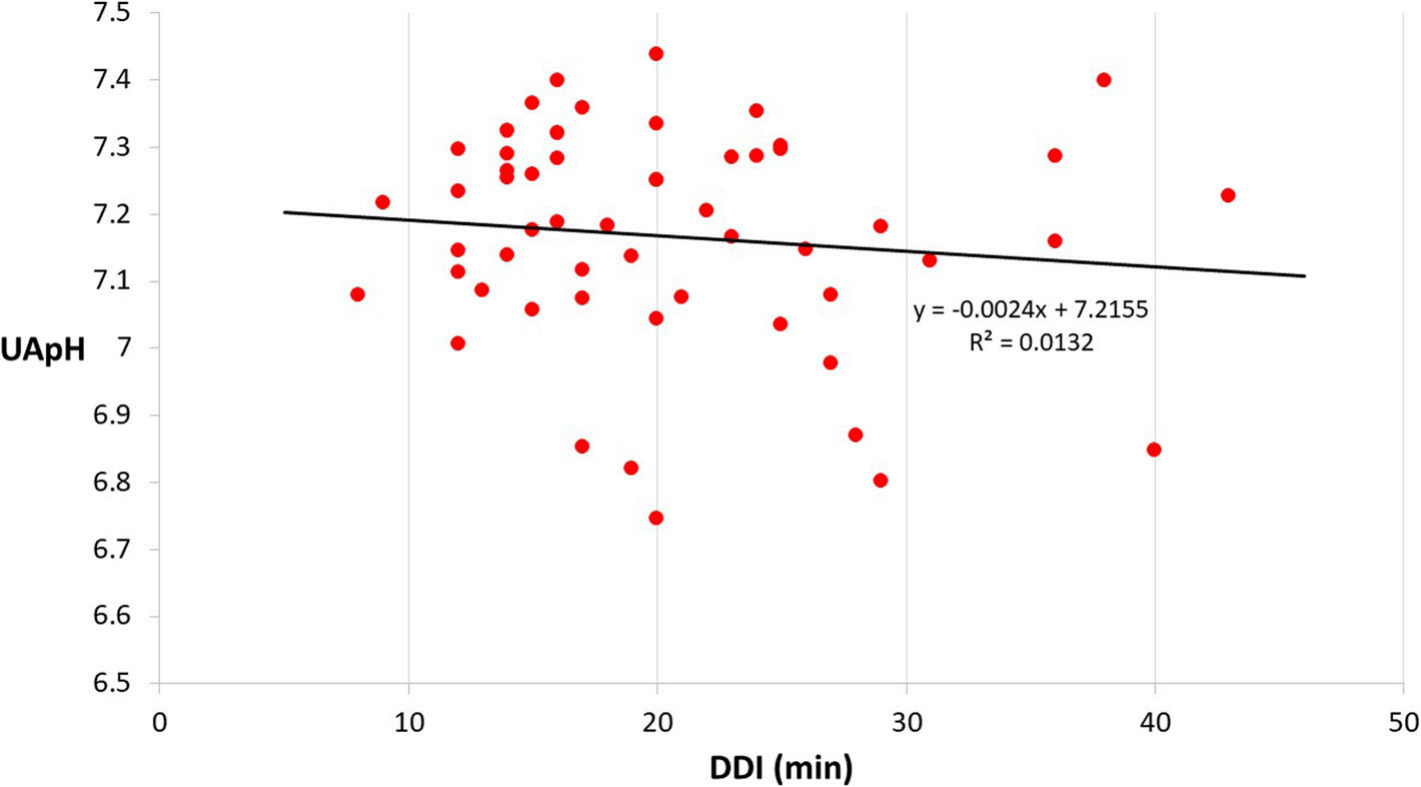
The relationship between decision to delivery interval and neonatal umbilical arterial pH. There were 59 urgent cesarean cases in the study years 2010 to 2019, including two cases of twin deliveries. Decision to delivery interval (DDI) was not determined in two cases, and neonatal umbilical arterial pH (UApH) was not recorded in four cases. Analysis of a total of 55 cases suggested a slight inverse correlation between DDI and UApH

Unlike the substantial decrease in elective and emergent cases, we have experienced a somewhat variable but non-declining number of urgent cesarean deliveries each year (Fig. 6). Overall, non-reassuring fetal status was responsible for many of urgent cases (Table 3). The decision to delivery interval (DDI) differed depending on the case, with a median value of 19 min (IQR 15–25 min) in the years 2010–2019 (Table 4). The mean neonatal umbilical arterial pH (UApH) was approximately 7.20, albeit with the exception of 2010, 2011, and 2014, whereas the median 1- and 5-min Apgar scores varied throughout the study period (Supplementary Table 2). The relationship between DDI and UApH is illustrated in Fig. 7.

**Table 3:** Indications for urgent (category 1) cesarean deliveries performed under general anesthesia

	2010	2011	2012	2013	2014	2015	2016	2017	2018	2019
Total cases	3	3	3	7	10	4	8	9	4	8
Abnormal placentation	0 (0.0%)	0 (0.0%)	1 (33.3%)	2 (28.6%)	0 (0.0%)	0 (0.0%)	0 (0.0%)	0 (0.0%)	0 (0.0%)	0 (0.0%)
Non-reassuring fetal status	2 (66.7%)	1 (33.3%)	1 (33.3%)	1 (14.3%)	5 (50.0%)	2 (50.0%)	5 (62.5%)	7 (77.8%)	1 (25.0%)	3 (37.5%)
Placental abruption	0 (0.0%)	0 (0.0%)	1 (33.3%)	2 (28.6%)	3 (30.0%)	0 (0.0%)	2 (25.0%)	2 (22.2%)	0 (0.0%)	2 (25.0%)
Threatening uterine rupture	0 (0.0%)	0 (0.0%)	0 (0.0%)	0 (0.0%)	0 (0.0%)	1 (25.0%)	0 (0.0%)	0 (0.0%)	0 (0.0%)	1 (12.5%)
Maternal factors	1 (33.3%)	1 (33.3%)	0 (0.0%)	0 (0.0%)	1 (10.0%)	1 (10.0%)	1 (12.5%)	0 (0.0%)	0 (0.0%)	0 (0.0%)
Fetal factors	0 (0.0%)	1 (33.3%)	0 (0.0%)	2 (28.6%)	1 (10.0%)	0 (0.0%)	0 (0.0%)	0 (0.0%)	3 (75.0%)	2 (25.0%)

Maternal factors included eclampsia (one in 2010 and one in 2015), amniotic fluid embolism (one in 2011), hypertensive disorders of pregnancy (one in 2014), and impaired placental function (one in 2016)

Fetal factors included umbilical cord abnormality (one in 2011), umbilical cord prolapse (two in 2013 and two in 2018), and prolapse of fetal extremity (one in 2014, one in 2018, and two in 2019)

**Table 4:** Decision to delivery interval for urgent cesarean deliveries

Year	Number of cases	Minute; median (IQR)	Minute; mean ± SD
2019	8	17.0 (14.5–23.8)	19.8 ± 7.4
2018	4	15.0 (9.0–23.3)	15.8 ± 7.4
2017	9	15.0 (12.0–18.5)	15.1 ± 3.2
2016	8	22.0 (15.5–29.8)	23.1 ± 8.4
2015	4	18.0 (10.3–22.8)	17.0 ± 6.7
2014	10	20.0 (18.0–29.0)	23.3 ± 6.7
2013	7	20.0 (16.0–27.0)	22.9 ± 9.9
2012	3	16.0 (14.0–26.0)	18.7 ± 6.4
2011	3	25.0 (20.0–40.0)	28.3 ± 10.4
2010	3	(21.0)	(21.0)
Total	59	19.0 (15.0–25.0)	20.5 ± 7.7

Decision to delivery interval (DDI) is expressed as both median (interquartile range) and mean ± standard deviation (SD). DDI was not ascertained in two cases in 2010

### Neuraxial anesthesia for pregnancy with placenta previa

Recently, we have adopted the use of neuraxial anesthesia over GA for pregnancy with placenta previa. A total of 75 women with placenta previa without suspected placenta accreta (63 elective and 12 emergent) received neuraxial anesthesia for cesarean delivery in 2016–2018. The use of combined spinal-epidural anesthesia comprised the majority of these cases (88.0%). Internal iliac artery balloon catheters were placed in 6 cases prior to elective cesarean delivery.

During the same period, GA was administered in 13 women with placenta previa (9 elective and 4 emergent). The 9 parturients scheduled for elective cesarean delivery had preoperative placement of internal iliac artery balloon catheters. Total hysterectomy was additionally performed in 5 of the 13 cases. The average amount of intraoperative blood loss was 1729 ± 686 mL in 75 cases performed under neuraxial anesthesia, and 2682 ± 878 mL in 13 cases performed under GA (Supplementary Figure 1).

### Mortality and morbidity

We observed no anesthesia-related mortality during the entire study period. There was one case of amniotic fluid embolism associated with disseminated intravascular coagulation, which was followed by intraoperative massive hemorrhage. This case required emergent use of extracorporeal membrane oxygenation during the surgery. No case of difficult intubation was identified in which more than three attempts at laryngoscopy were made. We recorded 19 cases where the lowest oxygen saturation (SpO_2_) was below 90% following the rapid sequence induction of GA (Supplementary Table 3), but the total duration of desaturation was less than 1 min in every case. During the 10-year period, the parturients who received GA for cesarean delivery, including the one with amniotic fluid embolism, were discharged without any anesthesia-related morbidity.

### Anesthetic agents and muscle relaxants used for induction

Table 5 represents trends in sedative-hypnotic agents and muscle relaxants that were used at our institution for induction of GA for cesarean delivery. Although the use of thiopental and succinylcholine accounted for a great majority in 2010, the following years saw a significant shift towards the use of propofol and rocuronium. The combination of these agents has been common in recent years, especially since 2016. The use of vecuronium was occasionally seen until 2013, but it has rarely been used in the past 6 years.

**Table 5:** Trends in anesthetic agents and muscle relaxants used for induction of general anesthesia

Year	Sedative-hypnotic agents			Muscle relaxants		
	Propofol	Thiopental	Midazolam	Rocuronium	Vecuronium	Succinylcholine
2019	14 (93.3%)	1 (6.7%)	0 (0.0%)	15 (100.0%)	0 (0.0%)	0 (0.0%)
2018	15 (93.8%)	1 (6.2%)	0 (0.0%)	16 (100.0%)	0 (0.0%)	0 (0.0%)
2017	20 (95.2%)	1 (4.8%)	0 (0.0%)	21 (100.0%)	0 (0.0%)	0 (0.0%)
2016	19 (90.5%)	2 (9.5%)	0 (0.0%)	20 (95.2%)	0 (0.0%)	1 (4.8%)
2015	16 (72.7%)	5 (22.7%)	1 (4.5%)	21 (95.5%)	1 (4.5%)	0 (0.0%)
2014	12 (36.4%)	18 (54.5%)	3 (9.1%)	21 (63.6%)	1 (3.0%)	11 (33.3%)
2013	16 (44.4%)	20 (55.6%)	0 (0.0%)	23 (63.9%)	6 (16.7%)	7 (19.4%)
2012	13 (35.1%)	24 (64.9%)	0 (0.0%)	20 (54.1%)	7 (18.9%)	10 (27.0%)
2011	11 (28.2%)	28 (71.8%)	0 (0.0%)	14 (35.9%)	10 (25.6%)	15 (38.5%)
2010	4 (14.8%)	23 (85.2%)	0 (0.0%)	0 (0.0%)	4 (14.8%)	23 (85.2%)

## Discussion

### Main findings

Our review of obstetric anesthesia practice from 2010 to 2019 at our institution revealed a noticeable decline in the number of cesarean deliveries performed under GA, particularly in the recent 5 years. The reduction in obstetric GA was accompanied by a decrease in the number of parturients with placenta previa who received GA for cesarean delivery. On the other hand, however, urgent GA use for cesarean delivery was inevitable in some unplanned situations, indicating its importance throughout the study years.

### Previous trends in obstetric anesthesia

Generally, fewer than 10% of cesarean deliveries are carried out under GA, although this varies among different studies [8–10]. We note that, despite the standard set by the Royal College of Anesthesiologists [10], more than 10% of cesarean deliveries were performed under GA at our institution until relatively recently. The high proportion of GA use was partly attributed to a haunting case that we encountered in the first half of 2007. The parturient at 37 weeks of gestation who was diagnosed as having marginal placenta previa underwent an elective cesarean delivery under spinal anesthesia, but intraoperative conversion to GA was required due to unexpected massive hemorrhage (total blood loss 19,400 mL) that compelled the obstetricians to add abdominal total hysterectomy. In retrospect, the attending obstetricians and anesthesiologists were not fully aware of the possibility that she had placenta accreta, which cannot be readily detected prior to cesarean delivery. In the years 2007–2009, we experienced 7 cases of placenta previa with an intraoperative blood loss of more than 4000 mL, including the aforementioned case.

Consequently, administration of neuraxial anesthesia for cesarean delivery in the presence of placenta previa was discouraged following the 2007 experience, in case of intraoperative massive hemorrhage. In fact, it was previously reported that women with placenta accreta often needed to be switched to GA during surgery [11]. Back in those days, the implicit understanding at our institution was that it was customary for women with placenta previa to undergo GA for cesarean delivery. The parturients were, almost without exception, informed in advance from obstetricians that they would receive GA.

### Impact of the launch of our obstetric anesthesia team on clinical practice

In 2011 the Perinatal Center was officially established, and two years later, in 2013, one of our staff anesthesiologists took up his new duties with obstetric anesthesia. The year 2015 saw the launch of our obstetric anesthesia team, which served as a catalyst that provided team members with wide opportunities to discuss practices with obstetricians. Their successful attempts at identifying women with pregnancy-related risk factors prior to cesarean delivery led to a rethinking of the indications for GA in obstetric practice. There was a shift towards using GA only for parturients with placenta accreta, who have high risk of intraoperative massive hemorrhage, prolonged operating times, and the need for application of extended surgical procedures. This was largely responsible for the reduction in GA administration for pregnancy with placenta previa.

The overall use of GA in obstetric practice has been declining in concert with the increasing adoption of neuraxial anesthesia for cesarean delivery [12, 13]. However, there has been controversy and interinstitutional variation regarding the preferred option of anesthetic technique for cesarean delivery complicated by placenta previa [14, 15]. As part of optimizing the indications for GA in an effort to avoid GA-related complications, our current clinical practice encourages the use of neuraxial techniques in parturients without any abnormally invasive placentation. This trend, which has been driven by our obstetric anesthesia team, is in accordance with the evidence that neuraxial anesthesia can be used for cesarean delivery even in the presence of placenta previa [16–18].

Unlike those days when GA was virtually the only choice for pregnancy with placenta previa, we now administer GA only in parturients with high risk of hemorrhage, who were prenatally diagnosed as having placenta accreta with a high degree of certainty. This is related in part to the finding that the amount of intraoperative blood loss was larger in cases using GA. Our new strategy provides a major contribution to optimizing the selection of cesarean deliveries that deserve the use of GA.

### Activities of our obstetric anesthesia team

Since its formation in 2015, our obstetric anesthesia team has acted as a communication bridge between the department of anesthesiology and the department of obstetrics. The team consists of a team leader and several team members, who take turns staying in the obstetric ward on weekdays, assisting obstetricians in performing epidural anesthesia for labor and delivery. Once a week, the team members participate in a multi-disciplinary conference with obstetricians and neonatologists where the group shares detailed information on parturients with comorbidities. They discuss the individual’s current physical status, the preferred method of delivery and its timing, and the appropriate choice of anesthetic technique in terms of maternal and neonatal outcome. This has made it possible to keep the members informed of the most updated medical condition of high-risk parturients.

In close cooperation with the department of obstetrics, the team leader has been engaged in making clinical assessments of all women who undergo elective cesarean delivery. Obstetricians are encouraged to consult him about parturients scheduled for vaginal delivery with maternal or fetal complications, should they need support from our obstetric anesthesia team. Through discussion with the parturient and obstetricians in outpatient settings, the leader makes clear in advance what type of anesthetic technique to choose for scheduled cesarean delivery. This newly established system has benefited us even when the parturient should undergo an emergency cesarean delivery, as it has made it easier to quickly grasp her medical condition and problems.

### Decision to delivery interval

At any hospital that has an obstetric service, there is a constant need for being prepared for obstetric emergencies. When there is an immediate threat to the life of the mother or baby, a 30-min DDI has been suggested as the time frame within which delivery should be accomplished [19]. Although the recommended DDI is widely accepted as a pragmatic rule [20], whether it improves maternal and fetal outcomes remains unclear [21, 22]. Additionally, the feasibility of the 30-min rule in urgent settings has been questioned in some studies [23, 24]. During the study period, we have achieved a DDI of less than 30 min in most urgent cesarean deliveries, despite the difficulty in predicting when they would occur. This suggests the practicability of the recommended DDI at a university hospital, where the department of anesthesiology is expected to provide anesthesia for every surgical patient all day and night. It must be remembered that our success in maintaining the optimum DDI has also been owed to obstetricians, neonatologists, nurses, midwives, and all medical staff members, including those in the operating room, who work in cooperation with anesthesia providers in an endeavor to keep prepared for emergency or urgency for the sake of maternal and fetal safety.

Theoretically, a shorter DDI may save the distressed fetus from being exposed to intrauterine hypoxia [22]. In our analysis, the value of UApH varied markedly regardless of DDI, probably because each case had different reasons and situations for requiring an urgent cesarean delivery. Even in urgency, however, every possible effort must be made to ensure the earliest possible delivery, given that DDI was associated, though less directly, with the severity of fetal acidosis.

### Current trends and future concerns

Throughout the study period, difficulty with intubation was not common among our 267 parturients receiving GA. The minimum SpO_2_ was lower than 90% in 19 cases without prolonged oxygen desaturation after the induction of GA. This was partly because we are also committed to medical residency education, and the first attempt at laryngoscopy was made by a resident anesthesiologist.

Ensuring opportunities for training with obstetric GA is still a matter of concern. Some studies warn that trainee anesthesiologists may have little or no clinical experience with GA administration for cesarean delivery, as GA in obstetric practice has been largely replaced by the widespread use of neuraxial techniques [13, 25]. The observed trend towards fewer cesarean deliveries conducted under GA may foreshadow an eventual lack of exposure to obstetric GA during residency training. The situation could be even more serious, considering that anesthesia providers at our institution are continually expected to deal with unpredictable cases that necessitate urgent administration of GA. Awareness of the necessity for maintaining obstetric airway management skills should be raised.

### Anesthetic agents and muscle relaxants used for induction

Rapid-sequence induction using thiopental and succinylcholine was the previous standard for induction of GA for cesarean delivery [26]. More recently, propofol and rocuronium, which are commonly used for induction of GA in non-obstetric settings, have mostly replaced them, serving as the agents of choice for obstetric GA. Succinylcholine, a depolarizing neuromuscular blocking agent, has the fastest onset and shortest duration, and it remained the preferred choice for difficult maternal airway management, despite its fatal side effects including hyperkalemia and malignant hyperthermia [26]. However, the situation has changed since rocuronium, a non-depolarizing neuromuscular blocking agent, and sugammadex became available for clinical use. A high dose (1.0–1.2 mg/kg) of rocuronium creates excellent intubating conditions in 60 s [27], and 16 mg/kg sugammadex can provide a rapid reversal of profound neuromuscular blockade [28]. Manufacturing approval for sugammadex was granted in Japan in 2010, and there has been a notable shift towards the use of rocuronium since the year 2011. Given recent trends, our prediction is that the combination of propofol and rocuronium will continue to be the standard, replacing thiopental-succinylcholine induction.

### Limitations

Some limitations of our study should be mentioned. First, it is to be noted that there have been changes and improvements in anesthetic techniques and drugs that have created a paradigm shift over the years in the way obstetric anesthesia is provided. Our analysis highlights the maturing process where delivery of better perinatal care, including optimization of obstetric GA administration, was enabled through the contributions of our obstetric anesthesia team. Even then, we recognize that this achievement was driven in part by a growing body of evidence on obstetric anesthesia practice. Second, providing detailed information on comfort and recovery of parturients was difficult in the current study. The database used for our analysis deals mainly with obstetric anesthetic care and management in the operating room. Further studies will be needed to assess the effect of our renewed practice on obstetric women with respect to post-operative pain management, feasibility of early ambulation, and hospital length of stay.

## Conclusion

In summary, our retrospective analysis of cesarean deliveries performed under GA over the past 10 years has revealed reduced GA administration in obstetric women, which was associated with a decrease in the number of parturients with placenta previa receiving GA. In a successful attempt to identify high-risk parturients, our obstetric anesthesia team has made possible the optimized use of GA for cesarean delivery, contributing to a reduction in use of GA in obstetric settings. Continuing efforts are required to further strengthen the relationship with multi-disciplinary team members, so that we can take the initiative to play the role of peripartum physicians to ensure better obstetric management for women in pregnancy.

## Supplementary information

**Additional file 1: Supplementary Table 1.** The annual number of pregnancies with placental abnormalities at our institution. **Supplementary Table 2.** The neonatal umbilical arterial pH and Apgar scores at 1 min and 5 min for infants delivered via urgent cesarean deliveries. **Supplementary Table 3.** The occurrence of desaturation (SpO_2_ < 90%) following the induction of general anesthesia.

**Additional file 2: Supplementary Figure 1.** Total intraoperative blood loss in cesarean deliveries for placenta previa performed under general or neuraxial anesthesia in 2016–2018.

## Data Availability

The datasets used and analyzed during the current study may be made available from the corresponding author on reasonable request.

## Abbreviations

GA: General anesthesia
DDI: Decision to delivery interval
UApH: Umbilical arterial pH
