# Supplementary material for: A retrospective review of 10-year trends in general anesthesia for cesarean delivery at a university hospital: the impact of a newly launched team on obstetric anesthesia practice
Source: BMC Health Serv Res. 2020 May 13;20:421. doi: 10.1186/s12913-020-05314-2 (PMC7371464; doi:10.1186/s12913-020-05314-2)
Supplement: Supplementary file 1 — Additional file 1: Supplementary Table 1. The annual number of pregnancies with placental abnormalities at our institution. Supplementary Table 2. The neonatal umbilical arterial pH and Apgar scores at 1 min and 5 min for infants delivered via urgent cesarean deliveries. Supplementary Table 3. The occurrence of desaturation (SpO2 < 90%) following the induction of general anesthesia. [file 12913_2020_5314_MOESM1_ESM.docx]

**Supplementary Table 1. The annual number of pregnancies with placental abnormalities at our institution.**

| Year | Number |
| --- | --- |
| 2018 | 36 |
| 2017 | 33 |
| 2016 | 25 |
| 2015 | 24 |
| 2014 | 28 |
| 2013 | 24 |
| 2012 | 21 |

The number of women with placental abnormalities has recently been increasing, with previa accounting for the majority. Note that the source of the data was the department of obstetrics; there could be a difference between their database and ours.

**Supplementary Table 2. The neonatal umbilical arterial pH and Apgar scores at 1 minute and 5 minutes for infants delivered via urgent cesarean deliveries.**

| Year | UApH | Apgar score at 1 min | Apgar score at 5 min |
| --- | --- | --- | --- |
| 2019 | 7.25 ± 0.08 | 7.5 (5.3 – 8.0) | 8.5 (7.3 – 9.0) |
| 2018 | 7.13 ± 0.13 | 4.0 (1.3 – 8.3) | 7.0 (5.5 – 8.5) |
| 2017 | 7.19 ± 0.09 | 7.0 (3.5 – 8.0) | 8.0 (7.0 – 9.0) |
| 2016 | 7.26 ± 0.13 | 4.0 (1.0 – 8.0) | 7.0 (6.0 – 9.0) |
| 2015 | 7.26 ± 0.06 | 4.5 (1.8 – 5.8) | 6.5 (3.8 – 7.8) |
| 2014 | 6.99 ± 0.19 | 3.0 (2.0 – 7.0) | 6.0 (3.0 – 9.0) |
| 2013 | 7.21 ± 0.14 | 4.0 (1.8 – 8.0) | 6.0 (4.0 – 9.0) |
| 2012 | 7.29 ± 0.04 | 7.0 (0.0 – 8.0) | 8.0 (2.0 – 9.0) |
| 2011 | 7.04 ± 0.20 | 3.0 (1.0 – 8.0) | 5.0 (1.0 – 9.0) |
| 2010 | 7.01 ± 0.24 | 5.0 (1.0 – 7.0) | 7.0 (2.0 – 8.0) |

Neonatal umbilical arterial pH (UApH) is expressed as mean ± SD. Apgar scores are expressed as median (interquartile range). The value of UApH was not recorded in four cases during the study years.

**Supplementary Table 3. The occurrence of desaturation (SpO_2_ < 90%) following the induction of general anesthesia.**

| Year | Frequency of occurrence of desaturation |
| --- | --- |
| 2019 | 13.3% (2/15) |
| 2018 | 0.0% (0/16) |
| 2017 | 4.8% (1/21) |
| 2016 | 14.3% (3/21) |
| 2015 | 4.5% (1/22) |
| 2014 | 3.0% (1/33) |
| 2013 | 5.6% (2/36) |
| 2012 | 10.8% (4/37) |
| 2011 | 7.7% (3/39) |
| 2010 | 7.4% (2/27) |
| Total | 7.1% (19/267) |
