# Supplementary figures and images for: A retrospective review of 10-year trends in general anesthesia for cesarean delivery at a university hospital: the impact of a newly launched team on obstetric anesthesia practice
Source: BMC Health Serv Res. 2020 May 13;20:421. doi: 10.1186/s12913-020-05314-2 (PMC7371464; doi:10.1186/s12913-020-05314-2)

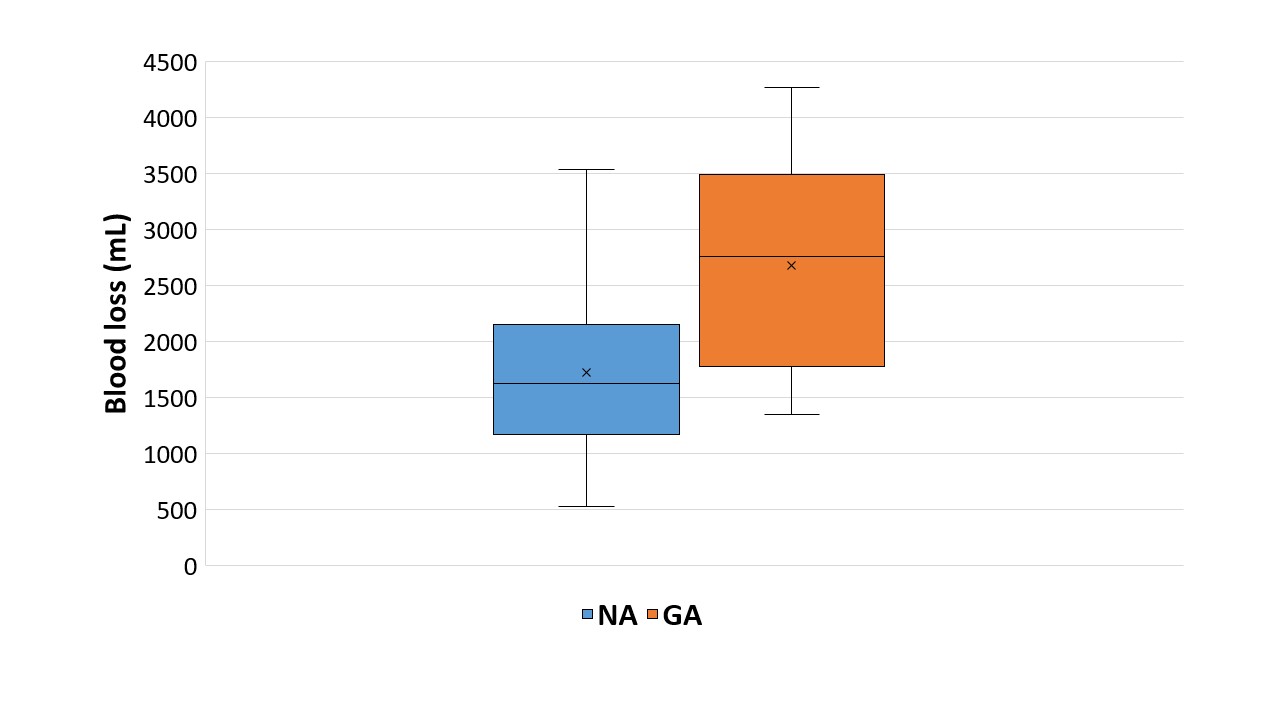

Supplement: Supplementary file 2 — Additional file 2: Supplementary Figure 1. Total intraoperative blood loss in cesarean deliveries for placenta previa performed under general or neuraxial anesthesia in 2016–2018. [file 12913_2020_5314_MOESM2_ESM.jpg]
